# “THIS IS NOT MY WAR”: MORAL CHALLENGES FACED BY MIGRANT HOME CARE WORKERS IN ISRAEL AT TIMES OF WAR

**DOI:** 10.1093/geroni/igae098.2945

**Published:** 2024-12-31

**Authors:** Liat Ayalon, Natalie Ulitsa

**Affiliations:** Bar Ilan University, Bar Ilan University, HaMerkaz, Israel; Bar Ilan University, Ramat Gan, HaMerkaz, Israel

## Abstract

The October 7 massacre had a tremendous impact on all persons residing in Israel, including the migrant workers community, which was specifically targeted by the Hamas terrorists. Subsequently, migrant workers have left the country in unprecedented numbers. This paper is based on interviews with 25 migrant home care workers who stayed in Israel during the Israel-Hamas war. Thematic analysis of in-depth interviews was conducted. Analysis revealed three main themes concerning moral dilemmas in the context of the war. The first moral dilemma concerned views of the war. Whereas migrant home care workers of Muslim background, attempted to distance themselves from the war, others expressed a strong identification with Israel. A second moral issue concerned the need to seek shelter within a short period of time, which often prevented older persons from reaching a safe room on time. This resulted in some workers leaving their care recipient during a missile attack to ensure their own safety. Workers attributed this behavior to specific instructions provided by the children of the care recipient. A third dilemma concerned the decision not to leave the country. Although many workers reported a wish to leave Israel following the war, this dilemma was largely resolved through the understanding that they have a financial debt that prevents them from returning to their home country. The findings highlight the challenges faced by migrant home care workers, who transitioned for financial reasons, but find themselves in a war situation with financial, emotional, and social commitments that challenge their loyalties.

